# The -137G/C Polymorphism in Interleukin-18 Gene Promoter Contributes to Chronic Lymphocytic and Chronic Myelogenous Leukemia Risk in Turkish Patients

**DOI:** 10.4274/tjh.2014.0126

**Published:** 2015-12-03

**Authors:** Serap Yalçın, Pelin Mutlu, Türker Çetin, Meral Sarper, Gökhan Özgür, Ferit Avcu

**Affiliations:** 1 Ahi Evran University Faculty of Engineering and Architecture, Kırşehir, Turkey; 2 Middle East Technical University, Central Laboratory, Department of Molecular Biology and Biotechnology, Ankara, Turkey; 3 Gülhane Military Medical Academy, Department of Hematology, Ankara, Turkey; 4 Gülhane Military Medical Academy, Cancer and Stem Cell Research Center, Ankara, Turkey

**Keywords:** IL-18, Chronic lymphocytic leukemia, Chronic myelogenous leukemia, Single nucleotide polymorphisms

## Abstract

**Objective:**

Interleukin-18 (IL-18) is a cytokine that belongs to the IL-1 superfamily and is secreted by various immune and nonimmune cells. Evidence has shown that IL-18 has both anticancer and procancer effects. The aim of this study was to evaluate the relationship between IL-18 gene polymorphisms and susceptibility to chronic lymphocytic leukemias (CLL) and chronic myelogenous leukemias (CML) in Turkish patients.

**Materials and Methods:**

The frequencies of polymorphisms (rs61667799(G/T), rs5744227(C/G), rs5744228(A/G), and rs187238(G/C)) were studied in 20 CLL patients, 30 CML patients, and 30 healthy individuals. The genotyping was performed by polymerase chain reaction and DNA sequencing analysis.

**Results:**

Significant associations were detected between the IL-18 rs187238(G/C) polymorphism and chronic leukemia. A higher prevalence of the C allele was found in CML cases with respect to controls. The GC heterozygous and CC homozygous genotypes were associated with risk of CML when compared with controls. However, prevalence of the C allele was not significantly high in CLL cases with respect to controls. There was only a significant difference between the homozygous CC genotype of CLL patients and the control group; thus, it can be concluded that the CC genotype may be associated with the risk of CLL. Based on our data, there were no significant associations between the IL-18 rs61667799(G/T), rs5744227(C/G), or rs5744228(A/G) polymorphisms and CLL or CML.

**Conclusions:**

IL-18 gene promoter rs187238(G/C) polymorphism is associated with chronic leukemia in the Turkish population. However, due to the limited number of studied patients, these are preliminary results that show the association between -137G/C polymorphism and patients (CLL and CML). Further large-scale studies combined with haplotype and expression analysis are required to validate the current findings.

## Abstract (tr)

**Amaç:**

İnterlökin-18 (İL-18), İL-1 süper ailesine ait bir sitokin olup, bağışıklık sistemine ait olan ve olmayan çeşitli hücrelerden salınmaktadır. Yapılan çalışmalar, İL-18’in hem anti-kanser hem de kansere öncülük eden etkilere sahip olduğunu göstermiştir. Bu çalışmanın amacı, kronik lenfositik lösemili (KLL) ve kronik miyeloid lösemili (KML) Türk hastalarda İL-18 gen polimorfizmleri ilişkisini değerlendirmektir.

**Gereç ve Yöntemler:**

İL-18 polimorfizleri (rs61667799(G/T), rs5744227(C/G), rs5744228(A/G) ve rs187238(G/C)), 20 KLL ve 30 KML hasta ve 30 sağlıklı bireyde araştırılmıştır. Genotipleme, polimeraz zincir reaksiyonu ve DNA dizi analizi ile gerçekleştirilmiştir.

**Bulgular:**

İL-18 geninde, rs187238(G/C) polimorfizmi ile kronik lösemi arasında anlamlı bir ilişki belirlenmiştir. KML hastalarında kontrol grubuna göre, C allelinin daha yüksek olduğu bulunmuştur. Kontroller ile karşılaştırıldığında, GC heterozigot ve CC homozigot genotipleri KML hastalarında risk oluşturmaktadır. Ancak, C alleli sıklığı kontrollere göre KLL olgularında istatistiksel olarak anlamlı değildir. KLL hastaları ve kontrol grubunun homozigot CC genotipi arasında anlamlı farklılık vardır ve bunun sonucu olarak CC genotipi, KLL hastaları için risk taşımaktadır denilebilir. Verilerimize dayanarak, KLL ve KML hastalarında, İL-18 geninde rs61667799(G/T), rs5744227(C/G) ve rs5744228(A/G) polimorfizmleri arasında anlamlı bir ilişki yoktur.

**Sonuç:**

İL-18 geninin promotor bölgesindeki rs187238(G/C) polimorfizmi Türk popülasyonunda kronik lösemi ile ilişkilidir. Ancak, yapılan bu çalışma, hasta sayısının sınırlı olması nedeniyle, -137G/C polimorfizmi ve hastalar (KLL ve KML) arasındaki ilişkiyi gösteren bir ön çalışma niteliğindedir. Mevcut bulguları doğrulamak için, haplotip ve gen ifade düzeyi analizleri ile birleştirilmiş daha geniş çaplı çalışmalara ihtiyaç vardır.

## INTRODUCTION

Interleukin-18 (IL-18) is a member of the IL-1 cytokine family [1]. It is secreted by various immune and nonimmune cells including T and B lymphocytes, activated monocytes, macrophages, Kupffer cells, natural killer cells, and Langerhans cells [2,3,4]. Evidence has shown that IL-18 has both anticancer and procancer effects [5]. IL-18 can stimulate natural killer cells and T cells promoting primarily Th1 responses, resulting in the elimination of tumor cells [6,7,8,9]. On the other hand, it has been reported that IL-18 is able to induce angiogenesis, migration, proliferation, and immune escape of tumor cells [10]. In models of hepatic melanoma metastasis the IL-18 blockade reduces the adherence of malignant cells by preventing IL-18 upregulation of vascular endothelial adhesion-1 molecule expression [11]. Higher expression levels of IL-18 are detected in different cancer types, such as gastric and breast cancer [12,13]. These results suggest that there is an association between the IL-18 gene and cancer risk, but this still remains controversial.

The IL-18 gene is located on chromosome 11q22.2-q22.23. A number of single nucleotide polymorphisms (SNPs) have been identified and investigated [14]. The IL-18 gene promoter -137G/C (rs187238) polymorphism is one of the most common SNPs, relative to the transcriptional start site, which may alter the expression of IL-18. This polymorphism can change the binding site of histone 4 transcription factor-1 nuclear factor and can have an impact on IL-18 gene activity [15].

Chronic myelogenous leukemia (CML) is a clonal bone marrow stem cell disorder characterized by the unregulated growth of mature granulocytes in the bone marrow and their accumulation in the blood [16]. The formation of the BCR-ABL fusion protein, which activates tyrosine kinase, plays a central role in the pathogenesis of CML [17]. Chronic lymphocytic leukemia (CLL) is the most common type of leukemia. CLL affects B cell lymphocytes that originate in the bone marrow, develop in the lymph nodes, and normally fight infection by producing antibodies [18].

It has been reported that malignant proliferation of leukemic cells is supported by a cytokine network surrounding these cells, produced partially by the cells themselves [19]. Elevated levels of IL-18 were observed in some leukemia patients, especially those with acute lymphoblastic leukemia and CML [20]. On the other hand, IL-18 receptor expression was reported mostly from CD19+ B cells and some CD8+ T cells [21].

The aim of this study is to evaluate the frequency of IL-18 gene promoter polymorphisms in Turkish CLL and CML patient groups and compare them with a control group in order to verify a correlation between the allelic variations and the risk of CML and CLL.

## MATERIALS AND METHODS

### Subjects

Twenty unrelated CLL patients and 30 unrelated CML patients diagnosed clinically at the Gülhane Military Medical Academy Department of Hematology and a control group of 30 unrelated healthy volunteers were randomly selected from different geographic regions of Turkey. The study protocol was approved by the local ethics committee of Gülhane Military Medical Academy and was conducted in accordance with the guidelines of the Declaration of Helsinki.

### Genotyping

The SNP in the promoter region of the IL-18 gene (SNP *g/c......rs187238; *g/t..... rs61667799; *c/g.......rs5744227; *a/g....rs5744228) was sequenced was determined by sequencing method. Genomic DNA was isolated from the peripheral blood by standard phenol-chloroform procedure. The genotyping of polymorphisms was performed by polymerase chain reaction and DNA sequencing analysis. A 446-bp fragment was amplified using specific primers (forward: 5’-CCAATAGGACTGATTATTCCGCA-3’ and reverse: 5’-AGGAGGGCAAAATGCACTGG-3’). Amplification was carried out on a Bio-RAD PCR system in 50 µL of reaction mixture containing 10 mM dNTPs, 25 mM magnesium chloride, 5 pmol each of forward and reverse primers, 2.5 U of Taq DNA polymerase, 10X PCR buffer, and 50 ng of genomic DNA. The PCR cycling conditions consisted of an initial denaturation step at 95 °C for 5 min, followed by 30 cycles of 94 °C for 1 min, 60 °C for 1 min, and 72 °C for 1 min, with a final extension step at 72 °C for 5 min. PCR products of 446 bp were then separated by 2% agarose gel electrophoresis at 120 V, stained by ethidium bromide (0.5 µg/mL), and visualized under a UV transilluminator. Single-pass sequencing was performed on each template using the forward primer. Cycle sequencing was carried out using the BigDye Terminator v.3.1 Cycle Sequencing Kit (Applied Biosystems, USA) according to the manufacturer’s instructions. The fluorescence-labeled fragments were purified by sodium acetate-ethanol precipitation method. Samples were then resuspended in distilled water and subjected to electrophoresis in an ABI PRISM 3100 Genetic Analyzer (Applied Biosystems).

### Statistical Analysis

SPSS 16.0 (SPSS Inc., USA) was used for the statistical analysis. Allele and genotype frequencies of alleles and genotypes were obtained by direct count. Statistical significance was defined as p<0.05.

## RESULTS

**Figure 1:**
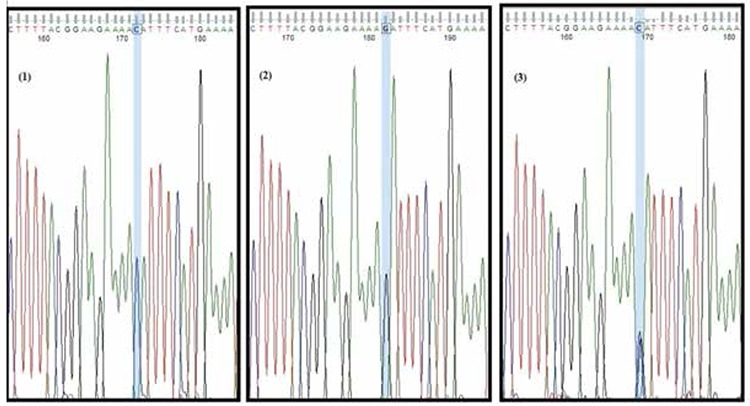
Representative chromatograms of sequenced PCR products, 1: homozygous mutant genotype (CC), 2: homozygous wild genotype (GG), 3: heterozygous genotype (GC).

Genotypes of the CML and CLL patients and the controls were determined by using DNA sequencing methodology for promoter polymorphisms. Figure 1 shows the chromatograms, which contain DNA fragments representing homozygous wild-type, heterozygous, and homozygous mutant genotypes (rs187238 G/C). The genotype and allele distributions of the controls versus CML and CLL patients are given in Tables 1 and 2, respectively.

Among the control group subjects, 77% were found to be homozygous for the GG genotype and 23% were heterozygous. There were no subjects with the CC genotype. The G allele frequency was 88% and C allele frequency was 12%. Among the CLL patients, 70% were found to be homozygous for the GG genotype, 10% were heterozygous (GC), and 20% were homozygous for the CC genotype. The G allele frequency was found as 75% whereas C allele frequency was 25%. Among the CML patients, 50% were found to be homozygous for the GG genotype, 37% were heterozygous (GC), and 13% were homozygous for the CC genotype. The G allele frequency was found as 68% whereas C allele frequency was 32%.

A higher prevalence of the C allele was found in CML patients with respect to the controls (p<0.05; Table 1). The GC heterozygous and CC homozygous genotypes were associated with the risk of CML when compared with the controls (OR: 1.9023; 95% CI: 0.6171-5.8636 for GC genotype and OR: 10.3585; 95% CI: 0.5326-201.4622 for CC genotype). These results indicate that individuals that are either heterozygous (GC) or homozygous for the CC genotype are associated with the development of CML in this Turkish population. However, prevalence of the C allele was not significantly high in CLL patients with respect to controls (p>0.05). There was only a significant difference between the homozygous CC genotype of CLL patients and the control group (p<0.05; Table 2). The CC homozygous genotype is associated with the risk of CLL when compared with controls (OR: 16.6364; 95% CI: 0.8428-328.3734). This indicates that homozygosity for the CC genotype was associated with the development of CLL in the studied patient group. There were no significant associations between the IL-18 rs61667799(G/T), rs5744227(C/G), or rs5744228(A/G) polymorphisms and CLL or CML.

## DISCUSSION

The -137G/C (rs187238) SNP in the promoter region of the IL-18 gene has a confirmed impact on gene activity and expression in tissues [15,22]. Some previous studies have suggested that IL-18 might act as a protumor factor in the progress of several tumors. The IL-18 protein has been shown to be overexpressed expressed in common skin tumors [1,23]. On the other hand, higher IL-18 levels both in the tumor region and in the serum were detected in metastatic gastric and breast cancer [12,13,24]. Furthermore, linkage between -137G/C and -607C/A polymorphisms of the IL-18 gene and progression of ovarian cancer and nasopharyngeal carcinoma was reported [25,26].

Based on 21 different studies, carriers of the variant C allele for -137G/C polymorphism were only reported to have a significant increased cancer risk compared with carriers of the G allele in nasopharyngeal carcinoma [27]. In the dominant CC genotype, some single studies suggested that the -137G/C polymorphism contributed to the susceptibility to certain cancer types, such as cervical [28], prostate [29], bladder [30], esophageal [31], and colorectal [32]. There are also several studies that concluded that there was no significant association between cancer and the -137G/C polymorphism [5,33,34]. Moreover, in one study, Monroy et al. found a significantly reduced cancer risk with the GC/CC genotype in Hodgkin disease [35].

These discrepant conclusions might be explained by ethnic differences since the studies that reported increased cancer risk were almost all carried out in Asians. On the contrary, a trend of reduced cancer risk was found in Caucasians [27].

To our knowledge, there are not many reports describing a comprehensive relation between -137G/C polymorphism and susceptibility to CML and CLL. In the present study, potential influence of the -137G/C polymorphism on both CLL and CML susceptibility was considered in a Turkish population. Our results showed a significantly increased risk in heterozygous (GC) and homozygous (CC) genotypes for CML. On the other hand, only the homozygous (CC) genotype is associated with the risk of CLL when compared with the controls. The results of this study may be important since there are not many reports showing the association of the -137G/C polymorphism with the risk of CML and CLL. However, there are several studies that showed dysregulated expression of IL-18 and/or IL-18 receptor in chronic B-cell lymphoproliferative disorders [36,37]. The dysregulated expression of IL-18 may be due to IL-18 gene promoter polymorphisms such as -137G/C. In addition, for this study, several limitations should be considered. First, the CML and CLL patient numbers were small. Second, haplotype analysis linking other IL-18 polymorphisms to IL-18 expression level may be necessary.

In conclusion, we demonstrate that IL-18 gene promoter -137G/C polymorphism is associated with CLL and CML in a Turkish population. However, due to the limited number of studied patients, these are only preliminary results that show the association between the -137G/C polymorphism and CLL and CML. Further large-scale studies combined with haplotype and expression analysis are required to validate the current findings.

## Figures and Tables

**Table 1:**
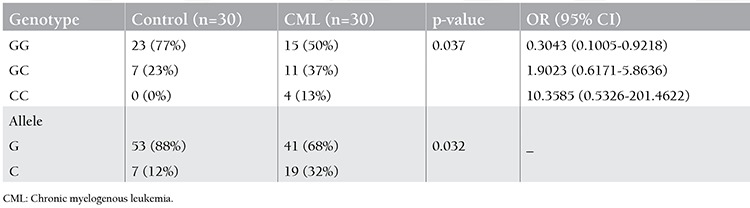
Genotype and allele frequencies of the -137G/C polymorphism in the control and chronic myelogenous leukemia groups.

**Table 2:**
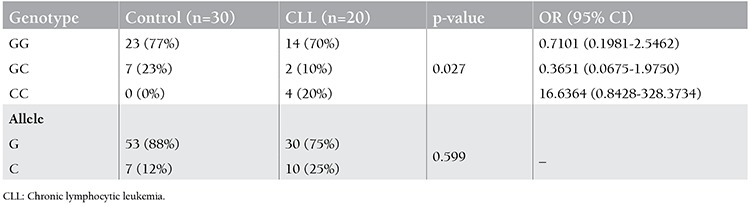
Genotype and allele frequencies of the -137G/C polymorphism in the control and chronic lymphocytic leukemia groups.

## References

[1] Dinarello CA (1999). Interleukin-18. Methods.

[2] Baxevanis CN, Gritzapis AD, Papamichail M (2003). In vivo antitumor activity of NKT cells activated by the combination of IL-12 and IL-18. J Immunol.

[3] Lebel-Binay S, Berger A, Zinzindohoué F, Cugnenc P, Thiounn N, Fridman WH, Pagès F (2000). Interleukin-18: biological properties and clinical implications. Eur Cytokine Netw.

[4] Tschoeke SK, Oberholzer A, Moldawer LL (2006). Interleukin-18: a novel prognostic cytokine in bacteria-induced sepsis. Crit Care Med.

[5] Liu JM, Liu JN, Wei MT, He YZ, Zhou Y, Song XB, Ying BW, Huang J (2013). Effect of IL-18 gene promoter polymorphisms on prostate cancer occurrence and prognosis in Han Chinese population. Genet Mol Res.

[6] Günel N, Coşkun U, Sancak B, Günel U, Hasdemir O, Bozkurt S (2002). Clinical importance of serum interleukin-18 and nitric oxide activities in breast carcinoma patients. Cancer.

[7] Dinarello CA (1999). IL-18: A TH1-inducing, proinflammatory cytokine and new member of the IL-1 family. J Allergy Clin Immunol.

[8] Gillies SD, Young D, Lo KM, Roberts S (1993). Biological activity and in vivo clearance of antitumor antibody/cytokine fusion proteins. Bioconjug Chem.

[9] Okamura H, Tsutsi H, Komatsu T, Yutsudo M, Hakura A, Tanimoto T, Torigoe K, Okura T, Nukada Y, Hattori K, Akita K, Namba M, Tanabe F, Konishi K, Fukuda S, Kurimoto M (1995). Cloning of a new cytokine that induces IFN-gamma production by T cells. Nature.

[10] Park S, Cheon S, Cho D (2007). The dual effects of interleukin-18 in tumor progression. Cell Mol Immunol.

[11] Dinarello CA (2000). Interleukin-18, a proinflammatory cytokine. Eur Cytokine Netw.

[12] Ye ZB, Ma T, Li H, Jin XL, Xu HM (2007). Expression and significance of intratumoral interleukin-12 and interleukin-18 in human gastric carcinoma. World J Gastroenterol.

[13] Eisse SA, Zaki SA, El-Maghraby SM, Kadry DY (2005). Importance of serum IL-18 and RANTES as markers for breast carcinoma progression. J Egypt Natl Canc Inst.

[14] Tsuboi K, Miyazaki T, Nakajima M, Fukai Y, Masuda N, Manda R, Fukuchi M, Kato H, Kuwano H (2004). Serum interleukin-12 and interleukin-18 levels as a tumor marker in patients with esophageal carcinoma. Cancer Lett.

[15] Gedraitis V, He B, Huang WX, Hillert J (2001). Cloning and mutation analysis of the human IL-18 promoter: a possible role of polymorphisms in expression regulation. J Neuroimmunol.

[16] (3 January 2014). Besa EC, Buehler B, Markman M, Sacher RA. Chronic myelogenous leukemia. In: Krishnan K (ed). Medscape Reference. WebMD. Retrieved ; available at.

[17] Yin CC, Abruzzo LV, Qui X, Apostolidou E, Cortes JE, Medeiros LJ, Lu G (2009). Del(15q) is a recurrent minor-route cytogenetic abnormality in the clonal evolution of chronic myelogenous leukemia. Cancer Genet Cytogenet.

[18] Harris NL, Jaffe ES, Diebold J, Flandrin G, Muller-Hermelink HK, Vardiman J, Lister TA, Bloomfield CD (1999). World Health Organization classification of neoplastic diseases of the hematopoietic and lymphoid tissues: report of the Clinical Advisory Committee meeting, Airlie House, Virginia, November 1997. J Clin Oncol.

[19] Zhang B, Ma XT, Zheng GG, Li G, Rao Q, Wu KF (2003). Expression of IL-18 and its receptor in human leukemia cells. Leuk Res.

[20] Taniguchi M, Nagaoka K, Ushio S, Nukada Y, Okura T, Mori T, Yamauchi H, Ohta T, Ikegami H, Kurimoto M (1998). Establishment of the cells useful for murine interleukin-18 bioassay by introducing murine interleukin-18 receptor cDNA into human myelomonocytic KG-1 cells. J Immunol Meth.

[21] Kunikata T, Torigoe K, Ushio S, Okura T, Ushio C, Yamauchi H, Ikeda M, Ikegami H, Kurimoto M (1998). Constitutive and induced IL-18 receptor expression by various peripheral blood cell subsets as determined by anti-hIL-18R monoclonal antibody. Cell Immunol.

[22] Kalina U, Ballas K, Koyama N, Kauschat D, Miething C, Arnemann J, Martin H, Hoelzer D, Ottmann OG (2000). Genomic organization and regulation of the human interleukin-18 gene. Scand J Immunol.

[23] Park H, Byun D, Kim TS, Kim YI, Kang JS, Hahm ES, Kim SH, Lee WJ, Song HK, Yoon DY, Kang CJ, Lee C, Houh D, Kim H, Cho B, Kim Y, Yang YH, Min KH, Cho DH (2001). Enhanced IL-18 expression in common skin tumors. Immunol Lett.

[24] Merendino RA, Gangemi S, Ruello A, Bene A, Losi E, Lonbardo G, Purello-Dambrosio F (2001). Serum levels of interleukin-18 and sICAM-1 in patients affected by breast cancer: preliminary considerations. Int J Biol Markers.

[25] Bushley AW, Ferrell R, McDuffie K, Terada KY, Carney ME, Thompson PJ, Wilkens LR, Tung KH, Ness RB, Goodman MT (2004). Polymorphisms of interleukin (IL)-1alpha, IL-1beta, IL-6, IL-10, and IL-18 and the risk of ovarian cancer. Gynecol Oncol.

[26] Pratesi C, Bortolin MT, Bidoli E, Tedeschi R, Vaccher E, Dolcetti R, Guidoboni M, Franchin G, Barzan L, Zanussi S, Caruso C, De Paoli P (2006). Interleukin-10 and interleukin-18 promoter polymorphisms in an Italian cohort of patients with undifferentiated carcinoma of nasopharyngeal type. Cancer Immunol Immunother.

[27] Yang X, Qui MT, Hu JW, Jiang F, Li M, Wang J, Zhang Q, Yin R, Xu L (2013). Association of interleukin-18 gene promoter -607C>A and -137G>C polymorphisms with cancer risk: a meta-analysis of 26 studies. PLoS One.

[28] Sobti RC, Shekari M, Tamandani DM, Malekzadeh K, Suri V (2008). Association of interleukin-18 gene promoter polymorphism on the risk of cervix carcinogenesis in north Indian population. Oncol Res.

[29] Liu Y, Lin N, Huang L, Xu Q, Pang G (2007). Genetic polymorphisms of the interleukin-18 gene and risk of prostate cancer. DNA Cell Biol.

[30] Jaiswal PK, Singh V, Srivastava P, Mittal RD (2013). Association of IL-12, IL-18 variants and serum IL-18 with bladder cancer susceptibility in North Indian population. gene.

[31] Pan HF, Leng RX, Ye DQ (2011). Lack of association of interleukin-18 gene promoter -607 A/C polymorphism with susceptibility to autoimmune diseases: a meta-analysis. Lupus.

[32] Guo JY, Qin AQ, Li RK, Yang CM, Huang FD, Huang ZY, Guo HJ (2012). Association of the IL-18 gene polymorphism with susceptibility to colorectal cancer. Zhonghua Wei Chang Wai Ke Za Zhi.

[33] Sáenz-López P, Carretero R, Vazquez F, Martin J, Sánchez E, Tallada M, Garrido F, Cózar JM, Ruiz-Cabello F (2010). Impact of interleukin-18 polymorphisms -607 and -137 on clinical characteristics of renal cell carcinoma patients. Hum Immunol.

[34] Haghshenas MR, Hosseini SV, Mahmoudi M, Saberi-Firozi M, Farjadian S, Ghaderi A (2009). IL-18 serum level and IL-18 promoter gene polymorphism in Iranian patients with gastrointestinal cancers. J Gastroenterol Hepatol.

[35] Monroy CM, Cortes AC, Lopez MS, D’Amelio AM, Etzel CJ, Younes A, Strom SS, El-Zein RA (2011). Hodgkin disease risk: role of genetic polymorphisms and gene-gene interactions in inflammation pathway genes. Mol Carcinog.

[36] Singer MK, Assem M, Abdel Ghaffar AB, Morcos NY (2011). Cytokine profiling as a prognostic markers in chronic myeloid leukemia patients. Egypt J Immunol.

[37] Airoldi I, Raffeghello L, Cocco C, Guglielmino R, Roncella S, Fedeli F, Gambini C, Pistoia V (2004). Heterogeneous expression of interleukin-18 and its receptor in B-cell lymphoproliferative disorders deriving from naive, germinal center, and memory B lymphocytes. Clin Cancer Res.

